# Nanoparticle-Based Contrast Agents for ^129^Xe HyperCEST NMR and MRI Applications

**DOI:** 10.1155/2019/9498173

**Published:** 2019-11-22

**Authors:** Jabadurai Jayapaul, Leif Schröder

**Affiliations:** Molecular Imaging, Leibniz-Forschungsinstitut für Molekulare Pharmakologie (FMP), 13125 Berlin, Germany

## Abstract

Spin hyperpolarization techniques have enabled important advancements in preclinical and clinical MRI applications to overcome the intrinsic low sensitivity of nuclear magnetic resonance. Functionalized xenon biosensors represent one of these approaches. They combine two amplification strategies, namely, spin exchange optical pumping (SEOP) and chemical exchange saturation transfer (CEST). The latter one requires host structures that reversibly bind the hyperpolarized noble gas. Different nanoparticle approaches have been implemented and have enabled molecular MRI with ^129^Xe at unprecedented sensitivity. This review gives an overview of the Xe biosensor concept, particularly how different nanoparticles address various critical aspects of gas binding and exchange, spectral dispersion for multiplexing, and targeted reporter delivery. As this concept is emerging into preclinical applications, comprehensive sensor design will be indispensable in translating the outstanding sensitivity potential into biomedical molecular imaging applications.

## 1. Introduction

Molecular imaging is considered as the realm in which the spatial distribution of different cellular and biochemical parameters occurring at the molecular level is displayed using different detection modalities. These include magnetic resonance imaging (MRI), fluorescence imaging (FI), ultrasound (US), and positron-emission tomography (PET). They all have demonstrated their usefulness as a valuable (pre-)clinical molecular imaging tool in different contexts. Various aspects need to be considered for each individual application (abundance of the target of interest, synthesis of the reporter, desired spatial and temporal image resolution, radiation burden, translational aspects, etc.), and each modality has its own pros and cons. In this context, MRI serves as one of the routinely used clinical imaging techniques due to its unlimited penetration depth and its capability to generate high-contrast images of different soft tissues with sufficient spatial resolution. Although MRI has important capabilities that are vital for molecular imaging, it suffers substantially from a lack of sensitivity. Any detected macroscopic magnetization requires a large spin density and thus typically also a relative high concentration of contrast agents [1] that act on the detected bulk magnetization.

A plethora of MRI contrast agents have been reported throughout many preclinical studies to address the aforementioned sensitivity issue and to explore the options of “responsive” agents [2]. In spite of tremendous rise in generation of new MRI contrast agents, mostly gadolinium (Gd^3+^)-based coordination complexes have found a major use in clinics, followed by superparamagnetic iron oxide nanoparticles (SPIONs [3]) in many preclinical studies. The MRI-active center like Gd^3+^ nuclei and Fe^2+^/Fe^3+^ spin states in SPIONs are capable of relaxing water protons that are available in their immediate vicinity, thus causing accelerated recovery of longitudinal magnetization (positive contrast, *T*_1_ shortening) or enhanced decay of transverse magnetization (negative contrast, *T*_2_ shortening). The relaxation processes of Gd complexes are governed by inner and or outer sphere mechanisms, while SPION relaxations are elucidated through Brownian and Néel relaxation mechanisms, respectively. In clinics, Gd^3+^ coordinated to linear (e.g., DTPA) or macrocyclic (e.g., DOTA) chelators is utilized as *T*_1_ relaxation agents. However, the stability of such chelates, particularly of the linear ones, and the related product safety have recently been highly debated [4]. Release of toxic Gd^3+^ into systemic circulation, followed by its undesired accumulation in different tissues, is a major concern [5, 6]. Therefore, novel and more safe alternatives have been introduced in order to avoid Gd release *in vivo*. Although some Gd agents are currently considered as safe, still there is a growing concern over Gd toxicity and its undesired interaction with different endogenous substances [7]. As the number of MR relaxivity agents based on Gd complexes that have been successfully implemented for molecular imaging purposes is anyway limited, the search for alternative and safe agents that could perform well under challenging *in vivo* conditions is ongoing. Hyperpolarized agents are currently explored in many studies as they address the sensitivity issue and do not require (super-) paramagnetic substances acting on the bulk water magnetization. They allow direct detection of dilute spin pools in compounds other than water. Xenon biosensors are one class of hyperpolarized (hp) reporters. Despite their different mechanism of action, their specific design is worth being discussed in the context of nanoparticle agents known from ^1^H MRI.

Unlike Gd-based agents, SPIONs and other related nanoparticles come with a large set of parameters to tune their behavior and relaxivity performance. Owing to their tunable size, SPIONs might easily extravasate into the leaky interstitial space and vasculature of different tumors, thereby leading to a nonspecific accumulation in tumors via an enhanced permeation and retention (EPR) effect. The EPR effect is fostered by macrophages and the reticuloendothelial systems (e.g., liver and spleen). Beyond such nonspecific accumulation, numerous targeted iron oxide nanoparticle-based MRI contrast agents have also been reported for preclinical imaging of different cancer cells and tumors. The challenge with SPIONs and their ultrasmall version (USPIONs, 1–20 nm) is still that they cause a “passive” signal cancellation and cannot be activated for switchable contrast through the MRI pulse sequence. The latter aspect is a feature that came with the advent of CEST agents, *i*.*e.*, a class of contrast media that relies on chemical exchange saturation transfer. CEST uses the concept of transferring selectively saturated magnetization of exchanging nuclei from a dilute contrast agent pool to the abundant bulk (water) pool, thereby causing an amplified signal decrease in the detected pool. It works with both diamagnetic substances [8, 9] and paramagnetic ones [10]. Moreover, a nanoparticle formulation in terms of liposomes [11] provides many opportunities to address various applications. All CEST agents have in common that an “off-resonant” reference image is compared with an “on-resonant” image where a selective RF saturation pulse has been applied on-resonant with the spins in the CEST agent pool to induce the saturation transfer for activating the image contrast.

The design of CEST agents is an emerging field of research, and nanoparticle approaches can address certain aspects of this new class of reporters. Part of the challenge for achieving a strong ^1^H CEST effect is that the intrinsic *T*_1_ relaxation of the water pool counteracts the actively driven saturation transfer and thereby limits the achievable signal contrast. The system reaches a steady-state magnetization with a signal loss compared to the reference scan. This is more pronounced for systems where the saturation transfer clearly outcompetes the relaxation [12]. Achieving a measurable decrease of the bulk (water) pool often requires relatively high concentrations of the CEST agent (10^−4^–10^−1^ M [13]). Constructs that enable a more efficient implementation of the CEST effect, e.g., by grafting many exchanging CEST sites onto a nanocarrier, are highly wanted. While the design of the agent itself with regard to its number and efficiency of exchange sites is a critical aspect, the general nature of the observed spin system is of major importance, too. The most efficient way to observe saturation transfer is actually achieved with a starting magnetization that does not self-renew and thus “stores” all saturation transfer from the CEST agent. This is achieved by working with hp nuclei as demonstrated in the HyperCEST approach [14]. The pool that induces the saturation transfer can be rather dilute and opens up new perspectives for addressing previously inaccessible targets [15, 16]. The combination of CEST and hp nuclei is highly beneficial to overcome the intrinsic poor sensitivity of MRI. In this context, hp ^129^Xe NMR and MRI have emerged as a promising technique for attaining very efficient CEST reporters. The combination of spin hyperpolarization and CEST provides a total leverage of ∼10^3^–10^5^-fold sensitivity enhancement over the conventional detection of dilute, thermally polarized nuclei.

**Figure 1:**
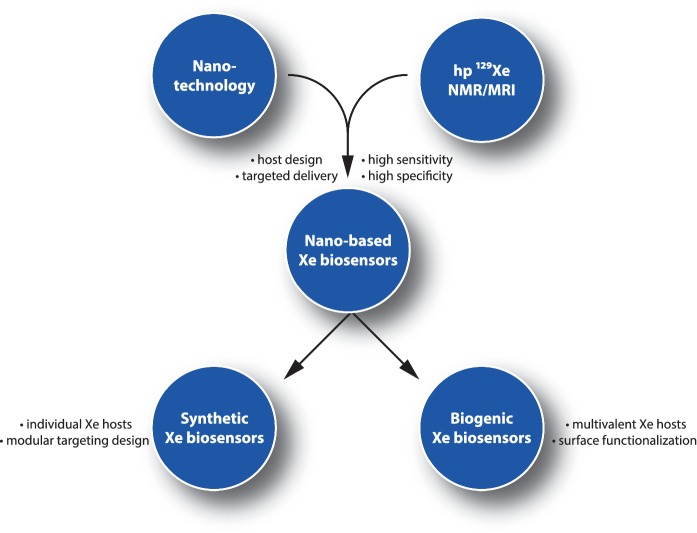
Concept of integrating nanotechnology into ^129^Xe NMR/MRI. The high specificity and sensitivity for hp ^129^Xe is paired with optimized host designs to improve Xe loading and gas exchange. The implemented solutions comprise both synthetic as well as biogenic host structures; the latter ones also include options for multivalent hosts that bind many thousand Xe atoms.

The role of nanoscience becomes eminent for the “functionalization” of hp Xe, particularly in the context of CEST applications (see Figure 1). As the noble gas is not part of any biologically relevant molecule or reporter that is eventually detected, host structures that trap Xe temporarily are necessary for making the HyperCEST approach possible. The large chemical shift range (∼300 ppm in solution) of ^129^Xe depending upon its chemical environment [17] is a key feature to design CEST pools with different resonance frequencies. Such dedicated host systems are necessary such that Xe can experience transient binding along with its in- and out-exchanges. Nanocarriers for Xe should provide a large loading capacity to affect many Xe atoms by the RF saturation pulse at any moment. Moreover, they should also come with a decent exchange rate to efficiently release the saturated nuclei into the surrounding bulk pool, followed by binding of fresh, not yet saturated nuclei.

Initial hosts were based on the macrocyclic compound cryptophane-A (CryA, [18]) that binds one Xe atom. It is still often used as the standard host in NMR/MRI with caged Xe. The hydrophobic CryA is functionalized by tethering the linkers, peptides, antibodies etc., through different handles that are available on it (e.g., CryA with a monoacid handle (CryA-ma) or CryA with a diacid handle (CryA-da) that shows separate CEST responses [19]). Its limited water solubility and the suboptimal CEST performance fostered the search for improved designs. Approaches based on nanotechnology can achieve better signal enhancement and enable different molecular imaging applications. Strategies are the integration of multiple Xe hosts per targeting unit or the use of hosts that have a larger loading capacity of many Xe atoms.

This review focuses on different nanosystems that are currently being evaluated as platforms for developing novel types of ^129^Xe biosensors. After a short introduction to the work with hp ^129^Xe as an NMR tracer, different aspects of combining nanotechnology with highly sensitive ^129^Xe NMR/MRI will be discussed. The performance of the nanotechnology-based Xe biosensors reviewed in this context will be compared to those of ^19^F and ^1^H NMR/MRI counterparts. Further, the challenges related to *in vivo* translation of nanoparticle-based Xe biosensors will be reviewed, followed by suggestions to improve their performance for future applications.

## 2. General Considerations for Hyperpolarized ^129^Xe NMR

### 2.1. Production of Hyperpolarized Xe

The noble gas ^129^Xe is typically hyperpolarized using spin exchange optical pumping (SEOP) [20–22]. In this process, the valence electron of vaporized Rb (emitted from a heated Rb droplet, melting point: 39.3°C) serves as a polarization precursor. The alkali metal is optically pumped on its D_1_ transition by a strong infrared laser (795 nm; ∼10^2^–10^3^ W cw power) that illuminates a pumping volume (10^2^–10^3^ mL) with circularly polarized light. In the presence of a static magnetic field (mT range) aligned with the laser beam direction, the polarized photons cause a transition from the ground to the first excited Rb electron state in combination with a spin flip according to the selection rules for dipole transitions. Collisional mixing causes equilibration between the spin sublevels of the ^2^*P*_1/2_ state. The system relaxes in a radiation-free manner by adding N_2_ as a quench gas (typically ∼10%) into the ground state with its two spin sublevels. As the pumping process selectively depletes only one of the latter ones, the system soon reaches a steady state with a strong overpopulation of the other electron spin states. The absorption profile of the Rb vapor is typically rather narrow compared to the emission of the laser diodes operating in cw mode. It is therefore common practice in many setups to add He as the major gas component (∼80%) and operate the system at a few bars over pressure to cause pressure broadening of the D_1_ transition for better photon absorption.

In a second step, the NMR-active ^129^Xe nuclei as part of the inflowing He/N_2_/Xe mixture are hyperpolarized through polarization transfer in a flip-flop process occurring with the prepolarized Rb atoms in the vapor state. Given sufficient laser power, the Rb electron spin is immediately repolarized and can serve other Xe atoms. Incoming Xe thus serves as a “sink” of polarization. The polarization transfer depends on the pressure and temperature conditions inside the pumping cell. Careful optimization can yield near-unity spin polarization with such a setup [23]. While a high laser power is *per se* useful to keep the Rb polarization high, it can become a challenge to manage the heat that is deposited into the N_2_ quench gas after the initial photon absorption through the Rb vapor. This heating can cause further vaporization of Rb and lead to a self-amplifying, detrimental process called “rubidium runaway.” This effect yields poor Xe hyperpolarization but can be controlled by appropriate temperature management inside the setup [19].

The production of hp Xe is possible using either a premix with a fixed Xe fraction or by implementing on-site combination of He, N_2_, and Xe prior to the polarizer gas inlet by using mass flow controllers in combination with a pressure controller at the outlet of the polarizer setup [24]. Many setups operate at Xe fractions of 2–5%, keeping in mind that higher Xe fractions require a stronger laser system to repolarize the Rb immediately after the spin exchange. The natural abundance of ^129^Xe is ∼26% and is thus sufficient for many NMR applications. However, *in vivo* applications can benefit from isotopically enriched ^129^Xe (∼80%) when signal averaging shall be avoided in the interest of time.

### 2.2. NMR Outside the Thermal Equilibrium and CEST Buildup

**Figure 2:**
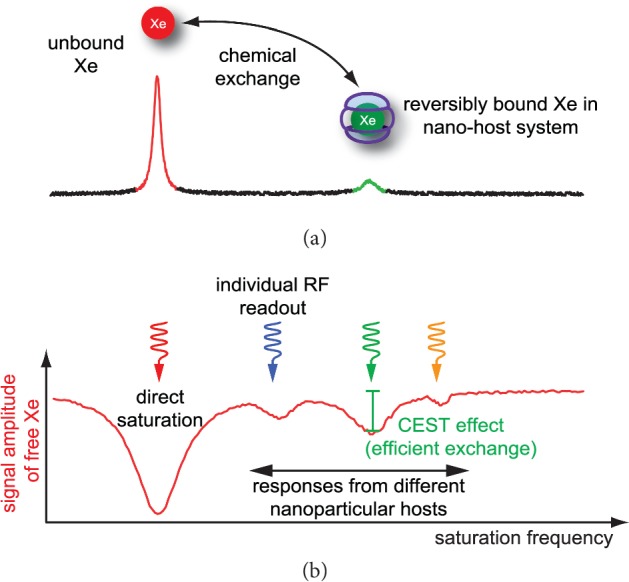
Concept of Xe HyperCEST detection. (a) Direct Xe NMR spectrum illustrating the abundant detected pool (in red) and the pool of reversibly bound Xe in a nanohost system (in green). (b) Applying the RF saturation pulse at different frequency offsets enables selective detection of different host structures in a CEST spectrum. The more efficient the Xe exchange is, the more pronounced will be the CEST response (as long as the exchange is not too fast to eliminate a separate chemical shift for bound Xe).

The hp spin system will eventually return to thermal equilibrium with an almost vanishing macroscopic magnetization. Hence, the hp gas provides its intense signal only for a limited amount of time and is often prepared for immediate delivery and detection. The relaxation process is governed by the *T*_1_ relaxation time that strongly depends on the local conditions affecting the Xe nuclei. Relaxation in gas phase is rather slow (*T*_1_ > 10 h; [25]) and is usually negligible as the typical HyperCEST application is fed with Xe directly from a continuously running SEOP setup. Once dispersed into solution, *T*_1_ of ^129^Xe drops to ∼100 s for water at 9.4 T or shorter values, depending on parameters such as the solvent and the protein content in solution. *In vivo* conditions cause further shortening of *T*_1_ and reflect the additional impact from paramagnetic species such as oxygen (during gas delivery via inhalation) and deoxy-haemoglobin in the bloodstream [26]. The Xe host used for HyperCEST must therefore provide conditions that ensure a buildup of saturation transfer that is faster than the intrinsic *T*_1_ relaxation of the bulk Xe. The saturation transfer is induced by applying a selective RF pulse onto the dilute pool of reversibly bound Xe. It is followed by detection of the bulk pool of unbound Xe. Different hosts provide different resonance frequencies for bound Xe and allow a selective readout (see Figure 2). Nanoparticles with a high Xe loading capacity are of particular interest as they can also provide a high total gas turnover rate to rapidly transfer the saturated magnetization from the nanocarrier into the bulk pool of detected Xe. The CEST method induces the depolarization of bound hp Xe upon applying the RF saturation pulse, and it depends on the saturation strength (*B*_1_) and duration (*t*_sat_), respectively. Both parameters, *B*_1_ and *t*_sat_, need to be optimized for a given exchange kinetics, and the specifications of a certain nanocarrier thus sometimes require adapting the NMR pulse sequence parameters.

Another cause of magnetization loss is from each readout pulse of the magnetization. In most cases, all magnetization is converted into transverse components, e.g., either by a single 90° pulse for spectroscopy or by a train of small flip angles for gradient echo imaging. Thus, no longitudinal magnetization is left for the following acquisition. Since HyperCEST requires at least two acquisitions (a reference scan and a saturation-activated scan), a redelivery of hp Xe is required. This extends to many gas deliveries for the acquisition of a whole CEST spectrum where the RF saturation pulse is applied at different frequency offsets. The delivery of Xe magnetization should thus be very reproducible. Optimization of the SEOP system for continuous flow operation where a certain fraction of the flowing gas mix is delivered into the sample can achieve high stability. A standard deviation of less than 0.5% has been demonstrated in a setup that was optimized for CEST measurements [19, 24, 27]. Given the starting magnetization is rather large and only one off-resonant measurement and one on-resonant measurement are acquired, it is possible to “share” the delivered magnetization for multiple acquisitions with a so-called smashCEST acquisition [28, 29].

### 2.3. Clinical Use of ^129^Xe MRI and Translation Potential

Hyperpolarized noble gases found their clinical applications in lung imaging, particularly in asthma and COPD studies [30, 31]. ^3^He and ^129^Xe are the most widely used species [32], but ^83^Kr has also been applied in some cases [33, 34]. Xe MRI is increasingly replacing the original He applications, also because of the recurring He shortage problem [35–37]. Like He, it produces images without any background signal from endogenous nuclei (e.g., water protons in tissue) and provides access to vital parameters such as gas diffusion and ventilation in lungs. However, Xe MRI applications go beyond gas phase imaging because this noble gas comes with a sufficient solubility in water [38] and thus also in blood and tissue [17]. This feature opens up possibilities for targeted contrast agents and stimulated the development of the first Xe biosensor [18]. The search for optimized Xe hosts happened independently from the development of Xe MRI in general, knowing that Xe detection from different tissues had already been demonstrated early on in small animal studies [39–41].

While chemical engineering continued introducing novel Xe host and sensing concepts, particularly for an expanding palette of molecular targets [42] and for improved CEST detection, hardware and pulse sequence development delivered significant progress for Xe MRI in human applications. The combination of (a) modern polarizers including high-performance laser diodes for a wider user community [43], (b) optimized RF antenna systems [44, 45], and (c) adapted readout protocols enabled novel studies of dissolved Xe in different tissues [46–48]. Imaging of the human brain has been demonstrated in the meantime [49–51] as well as Xe MRI of kidney perfusion [52]. Continuous advancements in this field demonstrate that the parallel development of Xe biosensors for ultrasensitive MRI has a high translation potential and warrants exploration of various nanocarriers for outstanding HyperCEST performance. A preliminary *in vivo* study [53] with CB6 as a Xe host under unfavourable conditions illustrated the applicability of HyperCEST in living organisms but also emphasized the urgent need for designing better host systems to fully translate the immense sensitivity boost that has been demonstrated with advanced Xe biosensors *in vitro*. As much as reproducible Xe delivery is important for *in vitro* studies, the conditions in living tissue also need to be stable enough to allow for reliable CEST detection. The conditions and requirements for detecting a saturation transfer buildup in live tissue during ongoing blood perfusion have been investigated in a previous study [54] which illustrated the high potential that nanocarriers loaded with many Xe atoms can have in particular.

## 3. Synthetic Nanosystems for HyperCEST Applications

**Figure 3:**
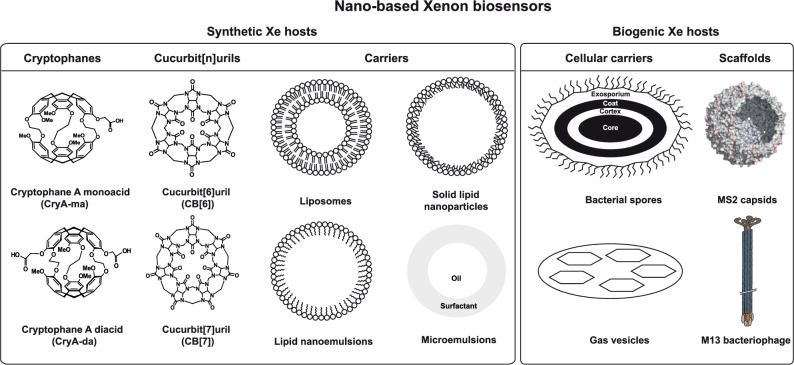
Comparison of different types of nanoparticle-based xenon biosensors classified as synthetic and biogenic host systems. The synthetic Xe hosts are categorized into macrocyclic-based nanocarriers (e.g., cryptophanes and cucurbit[*n*]urils) and non-macrocyclic-based nanocarriers (e.g., liposomes, nano- and microemulsions, etc.), respectively. The biogenic Xe hosts are typically delineated based on the cellular source from which they are engineered (e.g., bacterial spores and bacterial gas vesicles) or the scaffold platforms which they represent (e.g., MS2 capsids and M13 bacteriophage (adapted with permission from *Magn. Reson. Med.* 69(5): 1245–1252; copyright (2012) Wiley Periodicals, Inc. (bacteriophage) and from *Bioconj. Chem.* 27(8): 1796–1801; copyright (2016) American Chemical Society (MS2 viral capsids)) for installing multiple moieties that enable multivalent surface functionalization.

As mentioned above, the main purpose of Xe host systems is to convey a temporary, well resolved chemical shift onto reversibly bound Xe and to provide a binding moiety for a certain molecular target of diagnostic interest. The binding moiety depends on the specific application and is usually coupled separately. Regarding the gas binding aspect, nanocarriers can serve in two different ways: (a) in a scaffold-like approach where they carry multiple molecular host units (plus the binding moiety) or (b) simply as gas binding units themselves. In both cases, the nanoparticles serve as an attractive platform for generating highly sensitive ^129^Xe MRI contrast agents that are more efficient than the original HyperCEST implementation [14] with its minimalistic design (see Figure 3).

### 3.1. Functionalized Macrocycles as Nanosystems

Different macrocycles have been tested as hosts for transiently trapping Xe and for achieving guest exchange without any hindrance. Although many macrocycles exist, fine-tuning of the binding and exchange dynamics is not trivial. Cryptophanes were found to possess a cavity size that matches well with the Xe vdW volume. Initially, Xe binding to such cryptophanes was observed only in organic solvents with a dedicated peak for Xe in its bound state. Crytophanes provide a hydrophobic cavity formed by connecting two hydrophobic cyclotriveratrylene units (CTV) via three linkers of different lengths [55]. By simply varying the length of these linkers, different types of cryptophanes have been established.

Regarding cryptophane-A (CryA), three ethoxy alkyl linkers join the two CTV caps leading to formation of hydrophobic cavity (*V*_vdW_ = 95 Å^3^) that can host one Xe atom (*V*_vdW_ = 42 Å^3^). The three linkers determine the cryptophane's capability to exist in isomers and in different conformations, respectively. Upon binding of Xe, the Xe@CryA inclusion complex is characterized by a binding constant of ca. 8 × 10^2^ M^−1^ [56] (in water at room temperature) and a chemical shift of ∼62 ppm (Xe@CryA) compared to free Xe@solution (∼193 ppm), respectively [22]. Each CTV cap consists of three methoxy-substituted, methylene-bridged phenyls in which one of the methoxy groups in the CryA is replaced by a carboxyl or propargyl group to facilitate further functionalization with appropriate moieties. These methoxy groups play a major role in influencing the Xe binding, as shown by the observations that replacing them partly or as a whole with deuterium [57] and by having a different number of methoxy groups (3–12 groups per CTV) resulted in several conformations with altered overall Xe exchange dynamics [58]. Installation of two carboxyl groups onto CryA (CryA-da, see Figure 3) yields a Xe binding constant somewhat similar to that of CryA-ma albeit with a presumably slightly reduced exchange rate [59]. Meanwhile, many approaches have emerged to make CryA water soluble by either introducing multiple functional groups (e.g., acetate groups (tri- and hexafunctionalization) or by installing solubility enhancing amino acid sequences, respectively [60, 61].

Recently, water-soluble CryA derivatives (e.g., tris(triazole propionic acid)CryA (TTPC)) have been reported to form aggregates in water while they resolve into monomers upon binding to the protein targets [62]. These aggregations occur in sizes of tens to hundreds of nanometers at lower concentrations (nM–*μ*M) in water and or in buffers. It was hypothesized that such CryA nanoaggregates might appear due to the *π* − *π* interaction occurring between different phenyl rings of two CTV caps. However, these findings appear to be more specific and are being influenced by various conditions (e.g., temperature, ions, and solvent). Further evidence is necessary to prove that different xenon biosensors reported earlier indeed exist in such an aggregation state. Interestingly, inducing disaggregation by facilitating the binding between biosensor to a target molecule increased the CEST efficiency by ∼38%.

To make cryptophanes more water soluble, our lab investigated a generation 2 (G2) polyglycerol (PG) dendron grafted onto both sides of CryA-da [59]. At 13 *μ*M concentration, a CEST peak at −129.5 ppm with reference to the free Xe in solution peak was observed. Similarly, performing ^129^Xe HyperCEST MRI enabled localization of the dendronized CryA-da in par with the reference CryA-ma. It was found out that addition of dendrons onto the CryA-da did not significantly alter the Xe binding and exchange within the host cavity.

Cucurbit[*n*]urils (CB[*n*]) are another class of macrocycles for HyperCEST applications. These CB[*n*] are generated through the condensation of glycoluril and formaldehyde/paraformaldehyde under acidic reaction conditions [63]. Depending on the number of glycoluril repeating units, several types of CB[*n*] are well established, e.g., CB[*n*], *n* = 5, 6, 7, 8, 10. The two portals of these macrocycles are lined by the polar ureido carbonyl groups encompassing the hydrophobic interior cavity (see Figure 3). This makes them robust hosts for recruiting positively charged, hydrophobic, and neutral guests. CB[*n*] are reported to exhibit relatively high apparent binding affinity towards different guest molecules (∼1 × 10^5^ M^−1^). Earlier, CB[6] with improved solubility in water was reported to reversibly bind Xe in saline solution albeit with lower apparent affinity constant [64]. CB[6] solubility was enhanced by installing either hydrophobic (e.g., propane diurea and cyclohexyl) or hydrophilic moieties (e.g., mono- and perhydroxylation [65, 66]) at its bridging sites. Introduction of six cyclohexyl units at the CB[6] bridges promoted higher solubility and, subsequently, enabled the determination of Xe binding constant and exchange rates, respectively [67].

CB[*n*]s are widely employed in catalysis, drug delivery, nanoparticle functionalization, supramolecular chemistry, and in mediating different chemical reactions [68–73]. In terms of its usage in ^129^Xe NMR and MRI, there are few reports in which they have been successfully used for different applications. One such application is the generation of CB[6] based molecular rotaxanes that encapsulate a molecular thread (e.g., alkyl diamine) in which the CB[6] release is prevented by installation of appropriate stoppers (e.g., pyrene and adamantyl functionalized stoppers) at both ends of the molecular thread [74, 75]. Solubility of such stoppers was increased by introducing *β*-cyclodextrin (*β*-CD) that accelerated the rotaxane capture [76]. The assembled rotaxane prevented hp ^129^Xe from accessing the CB[6] cavity (“off” state, blocked for Xe), and it was successfully turned on after subjecting the rotaxane to ester hydrolysis. This process released the CB[6] from the rotaxane (“on” state, Xe accessible), thus promoting the binding of Xe to CB[6] cavity. In another application, CB[6] based rotaxane probes were developed for sensitive and selective detection of protease (matrix metalloproteinase-2, MMP-2) by ^129^Xe HyperCEST NMR [77]. To do so, a molecular rotaxane was synthesized using pyrene and TAMRA ((5,6)-carboxytetramethylrhodamine) as stoppers installed on both sides of the axle. The latter one was designed with a PLG-LAG peptide sequence specific for MMP-2 recognition. Two different pseudorotaxanes bearing a cleaved MMP-2 sequence were tested for the ability of CB[6] to slip off the rotaxane axle after the onset of protease-mediated peptide cleavage. Indeed, the evaluation indicated that CB[6] was easily released from the axle and became available for binding Xe to generate a HyperCEST response. After 24 h of cleavage reaction by MMP-2, Xe could access the CB[6] cavity to produce a 15% CEST effect. In yet another application, rotaxane probes were developed for sensing hydrogen peroxide using ^129^Xe HyperCEST NMR [78]. In this study, a sensitive and selective aryl boronic acid group was incorporated as cleavable cap on the rotaxane. The axle was built using a p-xylylenediamine moiety in order to have the lowest binding for CB[6]. Addition of two equivalents of H_2_O_2_ to the rotaxane probe capped with a maleimide unit induced a ^129^Xe HyperCEST response within 1 h and maintained maximum saturation of about 25%. Similarly, the detection limit of H_2_O_2_ in activated cancer cells stayed around 1 *μ*M, and the CEST response from Xe@CB[6] persisted even after 24 h albeit with significant peak broadening.

As a complementary approach for investigating enzyme activity, molecular CB[6] was utilized as host for following the progress of enzymatic action on a substrate using ^129^Xe HyperCEST. This has been demonstrated with L-lysine (Lys) that binds weakly to CB[6]. Being a substrate for lysine decarboxylase (LDC), its product cadaverine (Cad), however, binds strongly to CB[6] [79]. LDC activity eventually completely prevents Xe binding to CB6, and thus the HyperCEST response disappears in solutions of Lys that undergoes conversion through LDC. This can also be observed in time-resolved studies with accelerated encoding techniques [80]. The enzymatic assay performed using cell lysates and CB[6] led to significant broadening of the Xe@solution resonance without the indication of a dedicated Xe@CB[6] peak. Due to its limited solubility in water, CB[6] thus turned out to be not suitable for cellular studies and was replaced by CB[7] that can be present at higher concentrations [79]. However, treatment of macrophage cell lysates with CB[7] also leads to a significant increase in the line width of the Xe@solution saturation response. Detecting the dedicated HyperCEST response was eventually less efficient than detecting a change in magnetization transfer (MT) slightly off-resonant from the Xe@solution signal. Thus, a difference spectrum involving Xe@solution resonance was obtained by recording CB[7] spectra with and without LDC. The difference curve maximum (at ∼178 ppm, thus ca. 25 ppm offset from the Xe@solution peak) was used to perform the magnetization transfer ratio (MTR) imaging in order to precisely identify the enzyme-active compartment via an evident decrease in MTR signal.

Another emerging class of synthetic macrocyclic hosts for hp ^129^Xe are pillar[5]arenes which form a rigid cylindrical pillar-like structure as their repeating phenyl units are connected via the methylene bridges at the para position [81, 82]. Pillar[5]arene cavities are decorated with electron-rich groups that can assist in harboring electron-deficient and neutral molecules as guests. A water-soluble pillar[5]arene decorated with CH_2_COO–NH^4+^ end groups has been synthesized and has been shown to form a pseudorotaxane (1 : 1) complex with an alkane molecular thread (e.g., hexane) that serves as a co-guest in the presence of Xe [83]. Xe participating in such a ternary complex showed an upfield shift of more than 75 ppm compared to the reference (Xe in CDCl_3_). However, the chemical shift of Xe was shifted by more than 10 ppm downfield compared to the Xe@pillar[5]arene derivative in the absence of hexane as a co-guest. This suggests that Xe can be utilized as an NMR reporter to probe the information on the threading of the additional guest. As a follow-up study, our group has been screening Xe binding to several water-soluble pillar[5]arenes containing different counterions (e.g., Na^+^, NH^4+^, and tetramethyl groups) using magnetization transfer experiments [84]. Evaluation of the pillar[5]arene with Na^+^ counterions (340 *μ*M) using Xe NMR indicated no resolved signal for bound Xe; however, only a sharp resonance persisted for dissolved Xe. Conversely, pillar[5]arenes with sodium counterions (5 *μ*M) revealed a pronounced MT effect, *i*.*e.*, a direct saturation response FWHM of ca. 700 Hz at 295 K compared to parent pillar[5]arene and pillar[5]arenes with NH^4+^ counterions, respectively. The underlying difference in MT effect observed between parent compound and counterion-containing pillar[5]arenes might arise presumably from partial blocking of its portals by different counterions. This could cause a modulation of the Xe exchange rate. Aggregation of pillar[5]arenes with subsequent restricted access for Xe could be excluded. ^129^Xe MRI scans of the pillar[5]arene derived compound with Na^+^ counterions at 5 *μ*M host concentration demonstrated a superior performance compared to CryA: a reference sample with CryA-ma at 5 *μ*M yielded a CEST signal of 29% at 10 *μ*T saturation field strength, whereas the MT image showed a maximum signal change of 47%. Thus, MT detection using such pillar[5]arenes could be a promising alternative when limitations arise due to the specific absorption ratio (SAR) *in vivo*.

### 3.2. Incorporation of Cryptophanes into Nanoparticles (including Liposomes)

Hybrid nanosystems can be generated by coupling individual Xe hosts to a multimeric scaffold (nanosystem) to attain a high Xe CEST efficiency per scaffold. This strategy was pursued in various studies to improve the sensitivity even further since a rapid buildup of the CEST response is critical for applications with more challenging relaxation conditions. In an early version, dendrimers were evaluated as scaffolds for both encapsulation or installation of cryptophanes. For instance, fourth (G4) and fifth (G5) generations of polyamidoamine (PAMAM) dendrimers were shown to encapsulate 7 and 11 CryA in their hydrophobic interior [85]. These dendrimers with a biotin tag demonstrated approximately 8-fold gain in sensitivity upon binding to avidin in comparison to similar CryA-based biotin sensor without the dendrimer scaffold.

However, incorporation into such PAMAM dendrimers is limited, and a different concept based on a more efficient uptake of these hydrophobic hosts was explored. Since Xe is highly sensitive to its molecular environment, it was used to study the composition, fluidity, and domain fluctuations occurring in phospholipid bilayers [86–88]. Taking advantage of hydrophobic interactions between Xe, CryA, and the membrane, different Xe characteristics (e.g., exchange rate, partitioning, and detection of CryA in aqueous/lipid environment) in lipid environment have been evaluated by Xe NMR. Early on, CryA-da interaction with lipid vesicles (100–500 nm in diameter) of varying concentration showed that increasing the concentration of lipid vesicles increased the ratio of Xe@cage_lipid_ : Xe@cage_aq_ peak intensities [89]. This implies spontaneous partitioning of the cage into the lipid environment, and this incorporation into the lipid phase could be confirmed by fluorescence studies with giant unilaminar vesicles (GUVs) for which CryA had been coupled to fluorophores [90]. CryA in the lipid phase shows a resonance ca. 10 ppm downfield from Xe in CryA in aqueous phase. Such a characteristic downfield shift has also been observed in other studies where CryA had access to cell membrane material [91–93] and is helpful to identify cellular uptake of CryA compounds.

Following this observation of spontaneous self-insertion into phospholipid bilayers, it was realized that synthetic membrane vesicles such as GUVs can serve as nanoparticulate carrier systems for CryA (see Figure 3). The membrane composition has a significant impact on the observed HyperCEST effect. However, it is not possible to use the chemical shift information of CryA-bound Xe to distinguish different types of phospholipid constituents [90]. Instead, the exchange dynamics of Xe can be used to delineate differences between membrane compositions. Xe exchange in and out of the host is in general faster in lipid than in aqueous phase, but the membrane fluidity modulates this efficient exchange in a rather sensitive way.

This was first observed for the HyperCEST responses of two CryA-dye conjugates after mixing them with lipid vesicles made from different phospholipids (POPC, EYPC, and DPPC) [90]. The lipid vesicles generated in this study were of uniform size of 125 ± 30 nm. HyperCEST measurements revealed a large difference in signal intensities for different membrane systems in combination with no significant difference observed for fluorescence intensities. While the latter observation confirmed that the uptake of Xe hosts into the lipid phase was comparable, the first one pointed towards differences in the Xe exchange dynamics. Higher HyperCEST signal depletion was observed for POPC vesicles compared to other vesicles (e.g., EYPC and DPPC). This was attributed to a faster exchange rate in the more fluid POPC membrane with its kinked hydrocarbon chains. Increasing the temperature to 60°C exhibited a downfield chemical shift of all observed peaks (∼2–3 ppm) and an overall lower absolute signal intensity (ca. 50%). Performing HyperCEST measurements with increasing saturation time can thus be used to characterize the buildup of the saturation transfer for liposomal nanocarriers of different membrane fluidity. The acquired signal decay curves can be analyzed by an inverse Laplace transform to identify the characteristic decay constants. The method (called DeLTA for depolarization Laplace transform analysis) revealed ∼7-fold faster depolarization of exchanging Xe in POPC than in DPPC [86] and could also identify a variable amount of cholesterol in POPC liposomes that causes membrane stiffening [87]. Moreover, DeLTA results reflect pronounced fluctuations in apparent membrane fluidity that occur in the transition range of POPC/DPPC mixtures known from the formation of lipid rafts.

Besides this analytical aspect of CryA self-insertion into liposomal nanoparticles, the combination of individual Xe hosts with such nanocarriers has opened up new possibilities for designing highly efficient targeted HyperCEST agents. Different liposomes can be utilized as scaffolds for encapsulating a large number of hydrophobic CryA units. As an example, POPC-based large unilamellar vesicles (LUVs) were loaded with CryA where ca. 3800 CryA cages could be incorporated per liposome membrane [94]. The LUV structural integrity (diameter: 116 ± 2 nm) was still preserved at a [CryA] : [POPC] ratio of 1 : 20 while further loading with CryA caused disruption of the LUV membrane. The liposomes were then decorated in a second step including a targeting moiety through postinsertion of the cationic lipopeptide P2Rn (arginine (*R*) rich peptides). This decoration increased the observed liposomal size only marginally to 124.5 ± 3.4 nm, and the *R*-rich lipopeptides are internalized readily by the blood-brain barrier (BBB) lining cells via clathrin-independent and caveolin-independent endocytotic uptake mechanisms [95, 96]. Such a modular approach of CryA and lipopeptide insertion has the major advantage of circumventing elaborate synthesis steps that covalently couple the different functional units. Moreover, the toxicity of CryA + LUV and CryA + LUV + P2Rn was found to be significantly reduced compared to “naked” CryA when evaluating human brain microvascular endothelial cells (HBMECs) and human aortic endothelial cells (HAoECs) as control cell lines, respectively. Neither the incorporation of CryA cargo nor the positively charged P2Rn on the liposomes indicated any significant toxicity within the incubated biosensor concentration. By incorporating fluorescent labels into LUVs (rhodamine-labeled lipid (Rh-PE) for staining the membrane and carboxyfluorescein-labeled P2Rn for tracking the targeting units), it was found that both fluorescent tags colocalized within the HBMECs cells. Similarly, flow cytometry results indicated that LUV with P2Rn exhibited 7.4-fold higher uptake in HBMECs than control CryA + LUV liposomes. The specificity of CryA + LUV + P2Rn for HBMECs cells was further confirmed through ^129^Xe HyperCEST and MRI measurements. Such liposomes loaded with CryA and a targeting peptide enable highly sensitive cell labeling at particle concentrations in the range of ca. 1 pM [94].

### 3.3. Micro- and Nanoemulsions

Microemulsions are generally achieved by simply mixing two immiscible liquids (e.g., oil and water) along with a surfactant and, if required, with a co-surfactant. These microemulsions are thermodynamically driven relatively stable formulations that are produced without the need for applying any shear reaction conditions. Generally, the surfactant plays a major role in generating miscible formulation by forming a thin film or a monolayer at the interface between the different phases of two immiscible liquids. The overall stability of the microemulsions is influenced by temperature, pressure, and through the salinity of the aqueous phase, respectively. Exchanging Xe undergoing frequent transitions between the two emulsion phases experiences rapid changes in chemical shift. In this context, thermally polarized ^129^Xe NMR was utilized to establish the relation between resonance line widths and droplet diameter (1–9 *μ*m) of perfluorocarbon in an oil dispersed in water (o/w) emulsion [97]. Similarly, a ternary o/w microemulsion system was generated from pentaethylene glycol mono-*n*-dodecyl ether (C_12_E_5_), *n*-decane, and deuterium oxide (D_2_O), respectively [98]. Subjecting the above ternary o/w microemulsion to ^129^Xe NMR indicated a Xe residence time of ∼10 ns inside the oily core of the emulsion. Further, rapid Xe exchange occurring between the *n*-decane core and the continuum D_2_O via the C_12_E_5_ surface was validated through Xe NMR. Using a fast three-site exchange model for the microemulsion for analyzing the observed chemical shifts, the size of the oil droplets in microemulsion are determined through straightforward calculations. ^129^Xe NMR is also advantageous over light scattering-based techniques for studying dense microemulsion systems comprising a higher volume fraction of droplets since the actual droplet size determination using light scattering is often hampered by the interparticle interactions. However, Xe NMR is unable to provide any information about the shape of the oil droplets formed through microemulsion where complementary measurements of NMR relaxation and viscosity could furnish the required shape information [99–101].

#### 3.3.1. Solid Lipid Nanoparticles

Solid lipid nanoparticles (SLNs) are generated by utilizing solid lipids or a blend of solid lipids instead of liquid lipid (oil) while preparing the oil/water emulsion formulations. These SLNs exhibit particle sizes between 80 and 1000 nm and are readily dispersible in water or aqueous surfactant solutions [102]. Generally, SLNs are achieved through a melt emulsification, often followed by a hot or cold high-pressure homogenization process. SLNs are also prepared through precipitation of lipids using water-immiscible organic solvents or via semipolar water-miscible solvents, respectively [103]. Different lipids such as triacylglycerols, acylglycerols, waxes, and hard fats are utilized for generating SLNs. Unlike the classical SLNs described above, amphiphilic supramolecular building blocks instantaneously self-assemble to generate SLNs. Macrocycles such as calixarenes, cyclodextrins, and resorcinarenes are mainly evaluated for generating SLNs. These macrocyclic-based SLNs are interesting for encapsulating active ingredients (e.g., drugs) both in their cavity via molecular recognition and within the SLN matrix (see Figure 3). This facilitates high loading at multiple sites in addition to enhanced drug release profiles [104]. Since SLNs are nanocrystalline, the site distribution, structural information, and its mode of action have remained unexplored. Here, hp ^129^Xe NMR offers possibilities to study those aforementioned properties including site accessibility of SLNs.

Colloidal suspensions of amphiphilic calixarenes were generated by Friedel–Crafts acylation of the parent calix[4]arene. Subsequent freeze-drying resulted in monodispersed particles with 150 nm mean diameter. HP Xe NMR of amphiphilic calixarenes with chain lengths ranging between 6 and 16 carbons at room temperature showed four resonances at 0 ppm, ∼20 ppm, ∼80–130 ppm, and ∼190 ppm, respectively. These observed peaks are assigned to free Xe, Xe in the interparticle space, Xe residing in calixarene host cavity, and to Xe solubilized in the hydrophobic chains, respectively [105]. As the chain lengths of amphiphilic calixarenes increases, an increase in chemical shift of Xe bound to calixarene cavity resonance is observed. Xe NMR findings show that the hydrophobic chains (C_6_OH…C_12_OH) do not fold over or go into calixarene cavities, thereby allowing full access of the cavity to Xe. However, with longer chains (C_14_OH and C_16_OH), the accessibility of the calixarene cavity for Xe is somewhat limited. Evaluation of the absorption properties of calixarene with C_6_OH chain length using ^129^Xe MAS NMR in the presence of methylene chloride indicated two closely spaced peaks (*δ*_iso_ = 78.6 and 73.6 ppm) pertaining to Xe existence in two slightly inequivalent sites. Over time, the methylene chloride is released and the SLN cavity becomes more available for Xe binding as indicated through the rise in intensity of the peak assigned to bound Xe. Additionally, inclusion of methylene chloride to SLNs results in a phase transition between a state where hydrophobic chains self-include into adjacent calixarenes and another state where the chains are excluded, thereby making the host cavity available for further guest binding.

#### 3.3.2. PFOB Nanodroplets

This type of emulsion also requires mixing together two or more immiscible liquids in the presence of stabilizers (e.g., surfactants, co-surfactants, or co-solvents). Translucent nanoemulsions with droplet sizes below 100 nm can be obtained by choosing appropriate shear (*i*.*e.*, homogenizing) conditions (see Figure 3). These nanoemulsions are prone to coalesce over time, *i*.*e.*, the nanodroplets merge to form larger droplets. This effect can be mostly avoided by employing appropriate surfactants or emulsifiers.

Perfluorocarbon (PFC) nanoemulsions are utilized as ^19^F MRI [106] and ultrasound contrast agents [107] and also as vehicles for localized drug delivery [108], respectively. One such PFC is perfluorooctyl bromide (PFOB), which was earlier introduced as blood substitute since it solubilizes oxygen [109]. It was also proposed as a carrier for intravenous delivery of hyperpolarized ^129^Xe [97]. These PFOB characteristics promoted its evaluation as a highly sensitive Xe NMR contrast agent using ^129^Xe HyperCEST. Due to an increased solubility of hp Xe in PFOB, it was anticipated that an efficient Xe release from the droplets will cause a high contrast generation. PFOB was homogenized along with a poloxamer surfactant (Pluronic F-68) to yield perfluorocarbon-in-water nanoemulsions with narrow droplet size distributions (160–310 nm) [110]. The droplets were about 130 nm in size at 1 h after synthesis. It was shown that these droplets slowly grew over time leading to nanoemulsions with increasing average droplet diameters which occur due to slow coalescence after emulsification. Subjecting the PFOB nanoemulsions to ^129^Xe HyperCEST NMR yielded a dedicated peak for Xe bound to PFOB at ca. 100 ppm downfield with reference to free ^129^Xe gas signal at 0 ppm. With increasing droplet sizes, the CEST spectra indicated a decelerated Xe exchange, presumably occurring due to diffusion-limited Xe residence time inside the droplets. The PFOB nanoemulsion with droplet size of 310 nm was detected as low as 100 fM droplet concentration using hp ^129^Xe NMR with detectable Xe concentration of 94 *μ*M.

**Figure 4:**
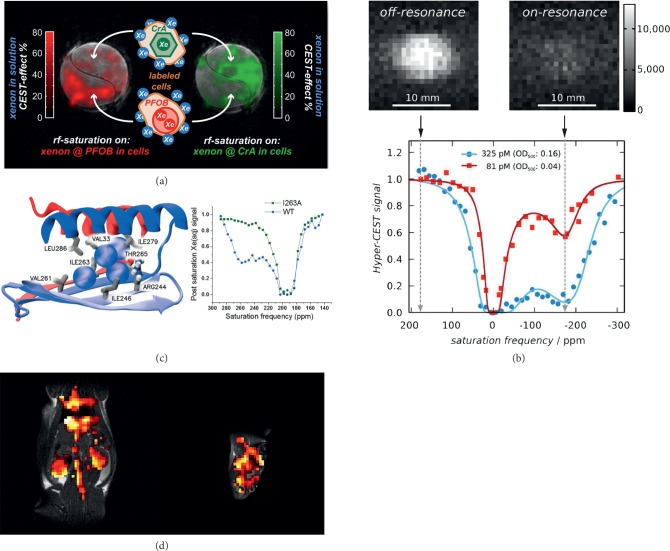
Examples of ^129^Xe HyperCEST applications. (a) Multichannel MRI labeling of cells using CryA-ma and PFOB nanodroplets as different Xe hosts with well-resolved saturation frequency offsets for selective CEST contrast (reprinted with permission from *Nano Lett.* 14(10): 5721–5726; copyright (2014) American Chemical Society). (b) Switchable ^129^Xe MRI contrast induced by bacterial gas vesicles (GVs) and the corresponding CEST spectrum at different nanoparticle concentrations (adapted with permission from *AIChE Journal* 64(8): 2927–2933; copyright (2018) American Institute of Chemical Engineers). (c) Sketch of the Xe binding site in *β*-lactamase (bla) and CEST spectra from the wild-type bla and the I263A mutation with suppressed CEST response (reprinted with permission from *Angew. Chem. Int. Ed.* 55(31): 8984–8987; copyright (2016) John Wiley & Sons Inc). (d) *In vivo* demonstration of HyperCEST MRI with CB[6] in a rat study showing contrast in the abdomen and the brain (reprinted under a Creative Commons Attribution 4.0 International License from *Scientific Reports* 7: 41027 (2017)).

As the resonance frequency of PFOB-bound Xe differs from that of CryA-bound Xe, these two hosts could be used in a multiplexing experiment for separate detection of different cell types (see Figure 4(a)) [111]. A similar multiplexing approach was first pursued with ^19^F NMR by labeling different cell types using high contrast generating PFOB nanodroplets [112–114]. For Xe MRI, this was implemented by performing multichannel HyperCEST NMR and MRI through nonspecific labeling of mouse fibroblasts (L929) using either CryA-ma or PFOB nanodroplets with confined sizes [111]. Unlike fluorescent dyes of different colours for multiplexing, Xe NMR comes with the additional feature of differentiating between Xe trapped in extracellular and cell-associated environment. For demonstrating the preserved chemical shift difference between the two Xe hosts upon cellular uptake, the same cell line was chosen for both nanocarriers, and a chemical shift difference of 40 ppm could be observed. This strategy eliminated the (presumably small) influence from different cell types (e.g., cell size and Xe uptake). The ^129^Xe HyperCEST spectra of cells incubated with 1.6 nM PFOB nanodroplets (diameter: 200 nm) for 18 h indicated a narrower PFOB-related CEST peak at 110 ppm compared to a signal at 120 ppm for Xe@PFOB in solution. ^129^Xe HyperCEST MRI of a cell suspension labeled with unfunctionalized PFOB nanodroplets (0.25 nM, 380 nm) revealed a higher HyperCEST effect than differently sized droplets with a mean value of 70% confined to PFOB-localized cells and saturated at the frequency of Xe@PFOB in cells. Additionally, using a bioreactor system for maintaining a perfused cell sample [92], we have shown that PFOB-labeled cells are viable, and they can be localized through 80 nM intracellular concentration compared to same cells loaded with a CryA-dye conjugate as another synthetic nanocarrier [111]. Although this demonstrates the cell labeling capabilities of PFOB nanodroplets, stabilization of PFOB with more robust surfactants is critical such that their overall nanosize will remain the same over time and during longitudinal studies.

A targeted version of the PFOB nanoemulsion with confined sizes might be useful in generating multimodal contrast agents (e.g., ^129^Xe and ^19^F NMR/MRI). To demonstrate the feasibility of targeted PFOB nanoemulsion-based multimodal contrast agents, PFOB nanodroplets were formulated using two emulsifiers such as Pluronic F-68 and Lipoid S75, respectively [115]. Additionally, a fluorescent tag (DiI) was tethered onto the phospholipid component of the nanoemulsion. The as-generated nanoemulsion was combined with cholesterol-labeled targeting peptide (e.g., Cls-PEG-RGDyc) such that cancerous cells from tumors expressing higher levels of *α*_*v*_*β*_3_ can be targeted. In this approach, a size control over the PFOB nanoemulsion was obtained by tuning the ratio between PFOB and phospholipids either via sonication or by extrusion of the final nanoemulsion. The ^129^Xe HyperCEST spectrum of untargeted PFOB nanoemulsion (UNE) indicated a CEST peak for PFOB-dissolved ^129^Xe at ∼106 ppm only when the particle size is larger than 250 nm at a concentration of about 5 pM. The targeting approach for PFOB nanoemulsion (RGD-nanoemulsion (RGD-NE)) was demonstrated by localization of lung cancer cells (A549) using ^129^Xe MRI (54% HyperCEST effect) over the nonspecific uptake of UNE by normal lung cells (WI-38) and through limited uptake of UNE by A549 cells (17% HyperCEST effect), respectively. These Xe HyperCEST MRI findings were complemented through similar contrast enhancement achieved using ^19^F MRI. Additionally, ^19^F MRI of an A549 tumor injected locally with RGD-NE showed a hot spot that helped to locate the tumor through its anatomical images acquired in this study. This work indicates that once stabilized with appropriate functionalizable emulsifiers, PFOB nanoemulsions can be effectively used as multimodal probes for detecting various cancerous cells and tumors. However, the *in vivo* stability and performance of these multimodal PFOB nanoemulsions has to be explored further.

## 4. Biogenic Nanocarriers for HyperCEST Applications

In general, synthetic nanocarriers are easily fabricated and functionalized using an appropriate targeting moiety for achieving molecular imaging applications with Xe NMR. However, the currently available macrocyclic-based rigid nanosystems can host only one Xe atom. In other cases like nonrigid nanocarriers (e.g., nanoemulsion/microemulsion and liposomes), many Xe atoms are transiently bound and Xe partitions quite efficiently into the hydrophobic layer of such nanocarriers. Except for liposomes, functionalizing such nonrigid nanocarriers on the surface is not straightforward. Hence, there is an ongoing search for systems which might act as a platform for tethering multiple hosts and specific tags for generating highly sensitive and selective Xe NMR contrast agents. The generation of different nanocarriers through bioengineering has thus attracted noticeable interest. These biogenic nanocarriers offer the possibility to anchor multiple units (e.g., hosts and tags) both at the surface and at the (sometimes hydrophobic) interior without any noticeable steric hindrance, respectively. Alternatively, in some cases, they can serve as multivalent Xe hosts themselves by providing hydrophobic cavities that are accessible to Xe. They also do not require any specific protecting coating like in the case of synthetic nanocarriers to avoid aggregation occurring due to their small size distribution. Once bioengineered, such nanocarriers are ready for tagging any molecules of interest onto them after chemically activating the respective amino acid residue handles. Depending upon the intrinsic properties of a certain biogenic nanocarrier, the extent of anchoring different moieties could boost the sensitivity and selectivity while addressing different molecular targets.

### 4.1. Viral Capsids

The MS2 viral capsid is a porous structure with an icosahedral symmetry comprising 180 monomers. These coat protein monomers spontaneously assemble into genome-free noninfectious capsids. The interior of such capsids could be conveniently assessed for modification without disassembly due to the 32 pores (each ∼2 nm wide) available on the surface (see Figure 3). This enables independent modification of both interior and exterior of the capsid. Regarding imaging applications, such capsids had been used earlier for decorating them with Gd chelates [116]. The search for scaffolds that could be decorated with CryA units therefore fostered an approach based on this concept. A mutation in MS2 was performed to introduce free cysteine on the capsid interior such that the linker with maleimide (Mal) could be tethered along with a taurine and an amine for facilitating CryA-ma coupling [117]. The extent of modification was estimated to be approximately 70%, corresponding to ∼125 copies of CryA-ma per assembled capsid called MS2CA. For referencing, ^129^Xe HyperCEST spectra of CryA (1 *μ*M) revealed the well-known peaks at ∼190 ppm (Xe@aq) and ∼60 ppm (Xe@CryA). Both a downfield and a slight upfield shift were observed for Xe@CryA and Xe@aq peaks, respectively, upon linkage of CryA to MS2. Additionally, a significant line broadening of Xe@CryA peak was observed in bioengineered MS2CA (∼5 kHz) compared to CryA alone (∼1 kHz). It was found that 7 nM MS2CA generated contrast similar to CryA at 1 *μ*M, and the lowest detectable MS2CA concentration by ^129^Xe HyperCEST was 0.7 pM.

Recently, such MS2 capsids were doubly modified using CryA-ma at the interior and targeting units (e.g., aptamers) at its exterior [118]. Here, a CryA-Mal linker containing five glutamic acids was coupled onto the cysteine present at the capsid interior. The coupling efficiency analysis revealed 60% modification of the MS2 which corresponds to ∼110 CryA per capsid. A fluorescent tag (Oregon Green maleimide) was installed on the interior for validating the cellular uptake by flow cytometry. Subsequently, the TD05.1 aptamer that targets membrane-bound mIgM markers that are highly expressed on lymphoma cells [119, 120] were modified using aminophenol groups at the 5′-termini. Further on, functionalized 5′-termini were conjugated to aniline side chains of the p-aminophenylalanine (pAF) residues on MS2 exterior via oxidative coupling [121, 122]. The specific binding of doubly modified MS2, *i*.*e.*, TD05.1-labeled capsids for targeting Ramos Burkitt's lymphoma cells expressing mIgM over Jurkat control cell line, was confirmed by flow cytometry. A broad Xe@CryA peak (at 57 ppm) was noted for Ramos cells after incubation with MS2-CryA-TD05.1 conjugates compared to no observable peak for Jurkat cells treated with the same incubation protocol. ^129^Xe Hyper-CEST MRI revealed a higher image contrast (∼50%) for the Ramos cell sample than the Jurkat cell sample.

### 4.2. Bacteriophages

Filamentous bacteriophages (e.g., M13 and fd types) are routinely used in phage display for identifying new epitopes (e.g., antibody fragments) [123, 124] and for expressing a wide variety of peptides and proteins [125]. Filamentous phages such as M13 and fd possess approximately five copies of minor coat proteins (p3, p6, p7, and p9) and varying levels of major coat proteins p8 Figure 3). The p3 sites are the principal locations where large protein and enzymes are inserted, while at the abundant p8 location, additional molecules (e.g., CryA host, dyes, and PEG spacers) can be amended to make them useful for nanoparticle nucleation [126], light collection [127], cell growth and differentiation [128], and drug delivery [129]. Regarding M13 usage for HyperCEST applications, the p8 protein was subjected to pyridoxal 5′-phosphate mediated biomimetic transamination reaction for introducing keto groups onto its N-termini [130]. Subsequently, a 5 kDa polyethylene glycol (PEG) unit, followed by a CryA-aminooxy peptide was installed on p8 of M13 using catalytic amounts of aniline. The resulting p8 coat proteins in M13-PEG-CryA biosensor were modified approximately with PEG-5k (760 copies, 28% efficiency) and with CryA (1050 copies, 39% efficiency). A ^129^Xe HyperCEST spectrum of M13 biosensor at 2.3 nM concentration revealed two peaks at 192 (Xe@aq) and 61.8 ppm (Xe@CryA@M13), respectively. In comparison, bound Xe in free CryA appears at 59.4 ppm. Statistically significant contrast (3.7% mean contrast) was achieved for M13 at a detection threshold of 230 fM M13, corresponding to 242 pM CryA.

As a follow-up study, the filamentous fd bacteriophage that displays single-chain antibody variable fragments (scFvs) on its minor coat proteins (p3) was utilized as a supramolecular scaffold for generating another highly sensitive ^129^Xe contrast agent [131]. The fd bacteriophage was engineered to express scFvs that are recognized by the epidermal growth factor receptor (EGFR) with high affinity [132–134]. The EGFR is a cell surface receptor which is overexpressed in different solid tumors (e.g., breast cancer) and is also linked to cancer progression [135, 136]. Similar to M13 phage, the fd-CryA biosensor (EGFR-CryA) was generated by applying similar chemistry, *i*.*e.*, pyridoxal 5′-phosphate mediated transamination, followed by oxime ligation with aminooxy-functionalized CryA-peptide catalyzed by aniline. The fd (4200 p8 copies) was modified (∼8%) with CryA units corresponding to ∼330 CryA per phage. Incubation of MDA-MB-231 (EGFR+) and Jurkat (EGFR−) cells with fd-CryA biosensors and staining for the bound phage using anti-fd bacteriophage antibody revealed the high binding specificity of biosensors to MDA-MB-231 cells. The specificity of EGFR-fd biosensor for EGFR receptor was proven by Xe NMR and MRI. ^129^Xe HyperCEST of MDA-MB-231 (EGFR+) cells incubated with this biosensor showed a response at 70 ppm (Xe@CryA) compared to no response for control Jurkat (EGFR−) cells. A HyperCEST contrast of 16.0 ± 9.4% was observed for EGFR+ cells compared to no contrast for EGFR− cells (1.4 ± 4.6%). Altogether, this targeted phage-based Xe biosensor binds to EGFR with high specificity *in vitro.*

### 4.3. Bacterial Spores

A spore is a highly resistant, dormant cell type produced by certain subset of bacteria in response to specific stresses such as, e.g., starvation [137]. In such conditions, spores are considered essentially as metabolically dormant [138]. However, they tend to emerge out of dormancy upon finding suitable growth conditions. Dipicolinic acid (DPA) analyte is an indication of spore formation, and it is present in all bacterial species. Therefore, it is impossible to discriminate between them using fluorescence and or by Raman spectroscopy via the dominant calcium DPA signals [139]. The detection of spores by ^1^H CEST MRI failed due to limited accessibility of endogenous H_2_O [140]. However, ^129^Xe HyperCEST was utilized to investigate bacterial spores (e.g., *Bacillus anthracis* and *Bacillus subtilis*) in solution via efficient saturation transfer [141].

Spores display a distinctive architecture involving series of concentric layers that contribute to resistance and other spore properties [142, 143]. The outermost layer is the “exosporium,” comprised of (glyco-)proteins, followed beneath (and separated by a gap) by the “coat” layer (see Figure 3). These two layers are found in both pathogenic and nonpathogenic species, and they play an important role in spore resistance. The *B. anthracis* strain with difference in exosporium/coat structure and variation in *B. subtilis* “coat” were distinguished in solution via differences in their ^129^Xe HyperCEST responses [141]. This observation has been assigned to changes in Xe diffusion occurring between spore interior (inner compartment) and bulk aqueous solution (outer compartment). Interestingly, the HyperCEST signatures allow distinguishing the bacterial species with and without exosporia, which normally remains undetected by other conventional methods. For example, wild-type *B. anthracis* (1.2 × 10^9^ cfu mL^−1^) revealed a Xe@spore peak at 196.3 ppm (broad) and a Xe@aq at 193.4 ppm, respectively. The broad line width of Xe@spore observed for different strains might be related to multiple weak binding and fast Xe exchanging sites available at the spores. Similarly, removing the coat layer in *B. subtilis* by mutation led to 2-fold HyperCEST contrast enhancement. This proves that spores lacking coat layers can be sensitively detected at 10^5^ cfu mL^−1^ by HyperCEST which indirectly reveals that inner layers (cortex, inner membrane, and core) serve as the Xe exchanging sites. Thus, HyperCEST serves as a valid tool in delineating morphologically similar spores with varied intrinsic characteristics through Xe exchange dynamics.

### 4.4. Gas Vesicles

Bacterial gas vesicles (GVs) are nanoscale hollow protein structures that enclose gas compartments (size range between 50 and 500 nm) (see Figure 3). They are expressed in different water-borne halo- and cyanobacteria. These GVs are generated by bacteria to control buoyancy and for facilitating migration to optimal depths in search of light and nutrients [144, 145]. The gas phase content of such GVs is in constant exchange with the gas molecules dissolved in the surrounding medium, thus promoting efficient gas exchange. Mostly, GVs comprise a highly conserved protein, GvpA, whose formation requires at least manipulation of eight genes located in the GV gene clusters [144]. It was anticipated that ^129^Xe will partition into GVs and that this will produce a distinct chemical shift for trapped gas atoms. In addition, the rapid exchange occurring between the GVs and dissolved gas should lead to the establishment of nanostructure-based genetically encoded HyperCEST reporters. The generation of GVs with different shapes and sizes from diverse bacterial species thus enables multiplexed Xe imaging.

GVs can be isolated from bacteria, e.g., from *Anabaena flos*-*aquae* (“*Ana*” GVs), by hypertonic lysis followed by purification using centrifugation-assisted flotation [24, 146]. *Ana* GVs (diameter ∼145 nm, length ∼250–1000 nm) at 400 pM concentration revealed a Xe@GVs peak at 31.2 ppm and Xe@aq peak at 195 ppm, respectively [54]. The binding of Xe to intact GVs was proven by observing no HyperCEST signal in the case of irreversibly collapsed GVs. A significant HyperCEST contrast of (33 ± 1.9)% was achieved for GVs by applying a saturation pulse of 0.8 s duration. A promising detection limit was demonstrated by a statistically significant contrast observed for GVs at 25 pM concentration that were saturated for 1.63 s. Additionally, ^129^Xe HyperCEST-based multiplexing was demonstrated through GVs with different shapes and sizes generated from diverse bacterial species, e.g., *Halobacteria* sp., NRC-1 (14.4 ppm), *Microcystis* sp., (30.6 ppm), and *E. coli* (51.4 ppm), respectively.

The targeting approach was demonstrated by functionalizing GVs generated from *A. flos-aquae* with biotin via the N-hydroxysuccinimide (NHS) linker installed on the lysine side chains of the GV surface. Then, a modular approach was followed to couple streptavidin-functionalized antibodies to biotin such that HER2 receptor-expressing cancer cells can be targeted using GVs. The HyperCEST images of HER2 breast cancer cell line (SKBR3) labeled with such anti-HER2 GVs showed a HyperCEST contrast of (78.53 ± 1.38)% compared to control Jurkat cells. These genetically encoded reporters indicated an *in vitro* sensitivity improvement of 10,000- and 100-fold in terms of molar concentration (25 pM) and protein mass (∼2.5 *μ*g ml^−1^) over ^1^H CEST genetic reporters, respectively [54]. It should be mentioned that GVs also function as switchable reporters for susceptibility-weighted proton MRI [147].

As a follow-up study, the different aspects of Xe interaction with GVs were quantitatively investigated. Contrary to Xe hosts that rely on chemical affinity (like CryA), an ideal CEST performance through simple physical partitioning of the dissolved gas into nanosized GVs could be demonstrated. This led to the concept of “elastic” HyperCEST contrast agent [148]. In this study, GVs of different size and shape were utilized that emerged from different bacterial sources. As a part of the elastic CEST concept, three GVs derived from *Anabaena flos*-*aquae* (Ana), *Halobacterium salinarum* (Halo), and *Bacillus megaterium* (Mega) with diameters (136 nm (Ana), 251 nm (Halo), and 72 nm (Mega)) and lengths (519 nm (Ana), 400 nm (Halo), and 250 nm (Mega)), respectively, were evaluated using quantitative HyperCEST (qHyperCEST). The qHyperCEST approach is based on a solution of the Bloch-McConnell equations [149]. It allows detailed quantification of the exchange parameters for reversibly bound hp Xe in different host systems [19, 56], including the bound Xe fraction, the exchange rate, and the relative chemical shift of bound Xe with respect to free Xe in solution. It was found that Halo and Ana GVs can host ca. 5800 and 4760 Xe atoms compared to Mega's lower hosting capabilities (ca. 890 Xe atoms) at 60 mbar total Xe pressure on top of the solution. This proved the direct relationship between the bound magnetization pool size and the size of the nanoparticles. The Mega GVs were used to investigate the elastic gas filling properties since these are the most stable GVs suitable for extended data acquisition times and high pressures. The qHyperCEST findings revealed that for different Xe gas total pressures, the CEST amplitude (given in % signal loss) remained stable and that the GV-bound Xe pool increases simultaneously in relation to the pool of free dissolved Xe. The Xe occupancy inside Mega GVs with ca. 0.65 aL volume corresponds to a Xe filling factor of ∼5% that matched well with the usage of 5% Xe fraction in the gas mix used to bubble Xe into the liquid. This partitioning behavior thus follows the ideal gas law and is determined by the Ostwald solubility coefficient, partial pressure, and temperature, respectively.

The observed CEST response is rather broad due to the relatively rapid exchange rate of Xe in and out of Mega GVs but still allows a selective activation of the CEST contrast compared to the reference scan (see Figure 4(b)). The HyperCEST effect was observed for Mega GVs in single scan MRI acquisitions with concentrations as low as 40 pM. Comparison of Mega GVs to CryA in terms of CEST performance after gradual pressure increase indicated that Mega GVs revealed a constant CEST contrast compared to a decrease of ca. 50% in CryA-induced contrast. This observation was attributed to a lack of elasticity in the CEST pool of CryA for self-adjusting the saturation transfer into the detection pool. The different GVs all act as high gas turnover (Xe host occupancy × exchange rate = gas turnover) hosts. They differ from other high Xe gas turnover complexes (e.g., Xe@CB[6]/CB[7]) by displaying constant CEST performance irrespective of the increasing free Xe pool size for increasing the overall signal quality. Importantly, recent progress has demonstrated heterologous expression of engineered gene clusters for encoding GVs in *E*. *coli* [150] and mammalian cells [151].

### 4.5. Gas-Binding Proteins and Enzymes

Early applications of Xe NMR included its use as a monoatomic biomolecular probe to study the structure of different proteins in solution, particularly with respect to its affinity for hydrophobic binding pockets. Although X-ray crystallography has been helpful in observing Xe binding sites in different proteins, it was unable to reveal the binding dynamics in both solid and aqueous conditions. To study such dynamics, NMR with hp ^129^Xe can provide important insights under different conditions. Proteins offer potential Xe binding sites such as surface exposed pockets, internal cavities, or void spaces and channel pores, respectively [152]. Generally, these sites might be lined with aliphatic, aromatic, or polar groups, and changes in side-chain conformations are reported to contribute to the creation of such Xe binding sites in proteins [153]. A polarization transfer from Xe to nuclei of residues near the Xe binding sites in proteins was anticipated early on to be beneficial for determining protein structures in cases where a crystal structure was unknown. Nonspecific Xe binding to the surface and cavities of different proteins, including metmyoglobin (metMb) methemoglobin (metHb), showed that Xe resides around 10–20 ps in the cavities without interfering with the protein's overall structure and stability [154]. However, high pressure and low temperature condenses Xe into a bulk state yielding no information about the Xe-protein interaction, and it also impedes the effective polarization transfer to nearby nuclei of the protein. Despite having several potential Xe binding sites, Xe occupancy into such cavities is mainly governed by the applied Xe concentration (e.g., 1–7 atm. pressure), temperature, and nature of the protein (native or denatured), respectively.

Further insights into protein structures were obtained by observing Xe-induced changes in chemical shifts of different nuclei such as ^1^H, ^15^N, and ^13^C linked in the vicinity of hydrophobic pockets in proteins. Such ^1^H and ^15^N NMR chemical shifts were used to map the residues affected by an artificially generated hydrophobic cavity in histidine-containing phosphocarrier protein (HPr) engineered from *Staphylococcus carnosus* by replacing isoleucine 14 with alanine (HPr(l14A)). The Xe-induced chemical shift for the wild-type HPr was about 0.05 ppm owing to an anticipated nonspecific interaction between Xe and the protein. However, the mutant version (HPr(l14A)) displayed a chemical shift of about 0.22 ppm suggesting specific interaction of Xe with the protein. Even higher chemical shifts of ca. 0.4–0.8 ppm were noted for amino acid residues lining up or being in close proximity to the engineered cavity of the mutant protein. This confirms that Xe-induced shifts observed throughout the molecule indicate structural rearrangements occurring due to the Xe binding in the hydrophobic cavity [155].

Following these initial observations, the Xe chemical shifts upon interaction with amino acids, peptides, and protein solutions under native and denatured conditions were studied in order to identify the role of different protein components on the overall nonspecific Xe interaction observed in proteins. This was represented by the concentration-normalized chemical shift denoted by *α* and expressed in ppm/mM. The molecular properties of different amino acids such as chemical functionality, structure, and charge are the factors that determine the Xe chemical shift *α* in solution. Denatured protein solutions show a linear dependence of protein size on *α*, meaning that it confirms the influence from diffusion-mediated interactions occurring under aqueous conditions. The changes in *α* are linked to protein-protein interface formation, changes in native state conformation, and protein denaturation. As the chemical shift is sensitive to different native state conformations of proteins (e.g., sugar binding proteins) [156], it is possible to assess conformational changes in the regime of ∼1 ppm/mM at protein concentrations of about 10 *μ*M [157].

The knowledge about Xe-protein interactions regained attention with the advent of HyperCEST. Several aspects of such early structural biology studies were revisited in order to elaborate the use of such gas-binding structures to define a system of exchange-coupled pools that could be used for CEST applications. Generally, Xe binding in different proteins is interpreted using “three-site” models that consider the following Xe environments: Xe in bulk solution (Xe@aq), Xe in hydrophobic cavities, and Xe at the protein surface. The Xe interaction at the protein surface is interesting for investigating proteins lacking well-defined cavities or pockets. The small chemical shifts observed for proteins in lyophilized state suggest that the signals emerge due to the surface-bound Xe in exchange with gaseous Xe. As an example, the accessibility of Xe in sperm whale metmyoglobin (MMb) was blocked by using the HgI_3_^−^ competitor resulting in reduced Xe line width and chemical shifts, respectively [158, 159]. However, performing the same experiment with horse MMb led to no complexation with HgI_3_^−^, proving the accessibility of Xe at its proximal cavity. Hence, the direct measure of Xe nonspecific binding at the protein surface is not straightforward and it does entirely depend on the precise conformation of the protein under investigation. Furthermore, the surface residues available at different proteins vary depending upon their hydrophobicity, charge, and polarity, and these characteristics regulate the Xe binding at the protein surface [160].

To demonstrate the feasibility of gas binding proteins (other than aforementioned GVs that represent a special case with their hollow structure) for HyperCEST applications, a single-protein TEM-1 *β*-lactamase (bla, 29 kDa) engineered from *E. coli* was developed as a genetically encoded (GE) HyperCEST reporter [161]. This enabled useful Xe MRI contrast in samples of both bacterial and mammalian cells. Bla is a small, monomeric bacterial enzyme that hydrolyzes *β*-lactam-based antibiotics, thus conferring antibiotic resistance onto its host. The gene expression in mammalian cell culture was studied by using bla activity-based fluorogenic substrates [162]. However, bla has been widely utilized in protein fragment complementation assays for investigating the protein-protein interactions both *in vitro* and *in vivo* [163]. Interestingly, bla possesses an allosteric site whose size and hydrophobicity may foster reversible binding of Xe. A “flooding” MD simulation revealed that Xe binds to the bla region existing between two terminal *α*-helices and the flanking *β*-sheet. Xe binding to the allosteric pocket of bla was observed to be a relatively slow process despite applying high Xe concentrations. A ^129^Xe HyperCEST spectrum of recombinant bla (80 *μ*M) indicated two peaks at 195 ppm (Xe@solution) and 255 ppm (Xe@bla), respectively. Both responses had a broad line width, thus indicating a fast Xe exchange occurring between the free and the transiently bound Xe state. The “molecular sensitivity” of bla was quantified at 0.1 *μ*M (2.9 *μ*g mL^−1^) where it produced a saturation contrast of 23%, *i*.*e.*, a value which is comparable to the previously reported GVs sensitivity detection limit [54]. ^129^Xe HyperCEST spectra of HEK293T/17 cells transfected with bla and those of nontransfected control cells indicated a slight shoulder at the Xe@aq peak. A low amount of transfected HEK cells (0.2 million cells/mL, ≃0.7 *μ*M·bla) was adequate for generating the saturation contrast of 0.13 ± 0.01 compared to minimal contrast observed for the control HEK cells. Although a bla-based ^129^Xe reporter is promising, still there is a need for sensitivity enhancement via the usage of isotopically enriched ^129^Xe [161].

Recently, it was attempted to characterize the Xe-bla interaction through X-ray crystallography, protein mutagenesis combined with X-ray based structural determination, and MD simulations such that the structural basis for the generation of the HyperCEST response can be elucidated. X-ray crystallography of bla indicated that it crystallized as a tetramer, and three Xe atoms (designated as Xe1, Xe2, and Xe3) were observed at similar (binding-)positions in all four bla molecules [164]. These three binding sites are comprised of predominantly hydrophobic protein side chains leading to no water occupancy. The structural refinement of the Xe@bla model indicated the highest average occupancy (68%) for Xe1, followed by 21% and 10% average occupancies pertaining to Xe2 and Xe3, respectively. Based on the predominant occupancy of Xe1, the association constant was estimated to be roughly 40 M^−1^ in the presence of 43 mM·MPa^−1^ Xe concentration in solution [165]. The structural dynamics of the Xe1 cavity was investigated in the presence of the competitive *β*-lactamase inhibitor tazobactam [166] using ^129^Xe HyperCEST. The CEST response assigned to the Xe1 cavity in the presence of the inhibitor indicated broadening of the Xe@bla peak (255 ppm), thus suggesting a faster Xe exchange occurring between the protein and solvent pool. Examining the Xe@bla crystal structure revealed no open pathway for Xe to transcend from the protein surface to the Xe1 cavity. This is made possible only through the dynamic fluctuations of the protein structure such that Xe can gain access to this buried Xe1 cavity. The X-ray crystallography and MD simulation-based findings indicated that bla possesses a high-affinity Xe binding site at Xe1 cavity and it could host three Xe atoms inside this cavity. To confirm the above results, single-point mutations were introduced to bla for perturbing the Xe1 affinity and or its exchange rates and pathways. The residues I263 and I279 were chosen for mutations as they regulate Xe access and likely affect the binding affinity to the Xe1 cavity. The residue I282 was mutated since it is necessary for establishing the van der Waals contacts with the Xe1 cavity. ^129^Xe HyperCEST spectra of bla mutants, *i*.*e.*, I263A and I279N, showed complete loss of CEST responses (see Figure 4(c)) while the protein denaturation occurring during the HyperCEST measurement did not contribute to the CEST loss. Conversely, the I282A and I263N mutants indicated an appreciable CEST contrast at 255 ppm with almost 50% lower intensity than the responses from WT bla. These different bla mutants confirmed that the bla's CEST signal at 255 ppm originates from the Xe1 binding site. Additionally, X-ray crystallography was performed on three mutants, namely, I263A, I263N and I263L, in order to determine the structural basis behind the generation of the ^129^Xe HyperCEST signal. The occupancy of Xe1 bound to I263L was 83% while low occupancies were observed for I263N (33%) and I263A (34%) mutants, respectively. Interestingly, the crystal structure of the three mutants revealed a new Xe binding hydrophobic cavity (designated as Xe5). The occupancies noted for Xe5 in different mutants I263L, I263N, and I263A are 22%, 28%, and 35%, respectively. The Xe5 binding site is located between Xe1 and Xe3 where the latter lies at the entrance of the hydrophobic channel. Based on the protein HyperCEST findings, it has been reported that the Xe binding does not always guarantee a useful MR contrast while the latter is mostly driven by the Xe exchange dynamics occurring between the protein and the solution pool. Further, MD simulations assessing Xe interactions with above mutants showed that even single-point mutations can highly impact Xe access to the protein cavity and in turn the observed CEST contrast [167].

Another genetically encoded small molecule-based ^129^Xe biosensor, namely, maltose-binding protein (MBP), was reported to detect nanomolar concentrations of maltose. MBP is a periplasmic protein encoded by the *malE* gene that serves as an initial receptor in the maltose/maltodextrin transport systems involved in Gram-negative bacteria [168]. Wild-type (WT) MBP binds maltose and other maltodextrins between two nearly symmetrical lobes transiting from an “open” (MBP_open_) to “closed” (MBP_closed_) conformation upon ligand binding [169]. ^129^Xe HyperCEST spectra of MBP at 80 *μ*M in the absence of maltose (MBP_open_) indicated only one response at 0 ppm corresponding to saturating free Xe in solution (Xe@aq). Contrarily, a CEST peak was observed for MBP at 95 ppm (Xe@MBP_closed_) in the presence of 1 mM maltose. This observed peak is 35 ppm further downfield compared to Xe@bla [161], suggesting that Xe@MBP_closed_ experiences a more hydrophobic environment. The lowest maltose concentration detected by MBP was 100 nM which translates to an observable saturation contrast of (5.0 ± 0.7)%. The maltose detection sensitivity was modulated through mutagenesis, *i*.*e.*, Ile-329 mutated to Tyr. This led to 32 nM as a ca. 3-fold improved detection threshold for generating a saturation contrast of (7 ± 1)%. The HyperCEST contrast evaluation of MBP in a cellular environment after expressing a MBP-GFP tag in *E. coli* resulted in a nearly 5-fold higher saturation contrast of ∼25% in the presence of maltose than *E. coli* expressing MBP in the absence of maltose. With these insights, the multiplexing approach using ^129^Xe HyperCEST was evaluated by utilizing both MBP and bla in the presence of maltose. The CEST spectrum of 27 *μ*M MBP and 80 *μ*M bla in absence of maltose indicated two peaks at 0 ppm (Xe@aq) and 60 ppm (Xe@bla), respectively. However, the presence of 1 mM maltose gave rise to three peaks at 0 ppm (Xe@aq), 60 ppm (Xe@bla), and 95 ppm (Xe@MBP), respectively. These multiplexing findings might pave the way for *in vivo* quantification of maltose via ratiometric analysis by using responsive (MBP) and nonresponsive (bla) HyperCEST agents. A superior MBP-based biosensor can be generated through mutations directed to CEST contrast enhancement and increased maltose binding. Additionally, rational mutagenesis will yield MBP variants with a broad range of ^129^Xe NMR chemical shifts similar to the palette of fluorescent proteins utilized for multiplexed cellular imaging [170, 171].

## 5. Discussion and Future Directions

### 5.1. Comparison to Nanoparticles Used in ^19^F and ^1^H NMR

When designing NMR reporters, their efficiency in terms of impacting magnetization for generating image contrast is of particular importance. This means they should either be detectable themselves as a strong magnetization or they should influence their surrounding magnetization as efficient as possible. Direct spin detection like in conventional ^1^H or ^19^F MRI is rather inefficient. This is due to the minute magnetization achieved from spin polarization at room temperature according to the Boltzmann distribution for spin-1/2 nuclei. Concentrations in the range of 10^−1^–10^0^ M are required, as illustrated by the detection limit of 10^12^–10^13^^19^F spins in a cell for the case of perfluoro-[15]crown-5-ether (PFCE) [172]. This molecule with 20 ^19^F atoms per unit has a density of 1.78 g/mL which translates to a minimum required uptake of ca. 0.4 pmole PFCE per cell (exemplary volume of 1 pL). This order of magnitude is consistent with the situation in conventional ^1^H MRI of the human body that relies on tissue water that makes up ∼70% of the body volume. Thus, the body-averaged ^1^H concentration is ∼80 M for high SNR, leaving room for detecting up to 100-fold lower concentrations under ideal conditions. However, many studies reported the necessity to go to much higher PFCE concentrations (>100 pmole per cell using cationic surfactant co-mixtures [173]) to achieve imaging without substantial signal averaging. In comparison, liposomal labeling of cells for Xe HyperCEST MRI like in reference [94] is rather efficient as it required a sample-averaged concentration of only 1 pM of the nanoparticles for labeling a suspension with 8 × 10^5^ cells/mL.

**Figure 5:**
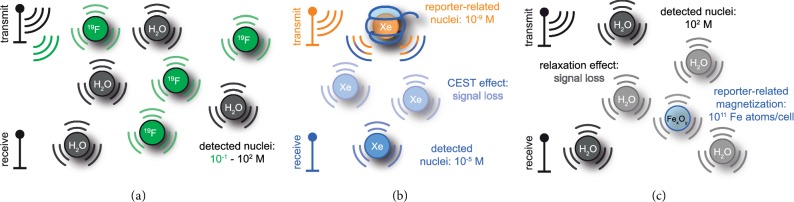
Comparison of different MRI approaches. (a) Conventional detection operates at an individual Larmor frequency to directly detect the nuclear magnetization of each spin species and requires high spin densities. (b) HyperCEST MRI manipulates reversibly bound Xe through a selective RF pulse and detects it on the separate Larmor frequency of free Xe. One reporter can impact many Xe nuclei. (c) SPIONs impact the bulk magnetization of nearby water; the RF operates only on the water Larmor frequency.

Nanoparticles for Xe HyperCEST fall into the category of indirect reporters which act on the bulk spin pool by “processing” a large number of the detected spins (see Figure 5). This is a rather efficient mechanism that also applies for superparamagnetic iron oxide nanoparticles (SPIONs). These particles are highly efficient for cell labeling applications in ^1^H MRI. One difference compared to Xe MRI is that HyperCEST requires the affected nuclei having direct access to the host, whereas the dipolar field of the SPIONs requires only a certain proximity of the diffusing water to experience the relaxation effect. Hence, the detected Xe has for sure been in the microenvironment of its functionalized host, whereas bulk water affected by SPIONs can be a separate (but nearby) compartment. SPIONs thus have a somewhat more “unspecific” radius of action.

### 5.2. Translation to *In Vivo* Applications

To overcome the toxicity concerns regarding Gd deposition, different types of contrast agents have been developed as contrast agents for preclinical MRI. In general, such nanoparticles are synthesized by pursuing one of the following synthetic strategies, namely, (1) incorporation of paramagnetic ions into the nanostructured framework (e.g., Gd_2_O_3_, MnO, Mn_3_O_4_, Dy_2_O_3_, NaGdF_4_, and ZnFe_2_O_4_ [174–176]) or by (2) postfunctionalization of the nanoparticles with a lanthanide complex (e.g., doping the nanoparticle scaffolds made of Si, Au, micelles, or quantum dots using DTPA and DOTA [177–179]). This approach of fabricating nanoparticles comprising paramagnetic ions might provide a control over its size, shape, composition, stability, pharmacokinetics, and biodistribution, respectively. Even though different lanthanide oxide-based nanoparticles help to reduce the toxicity burden compared to free Gd and other lanthanide complexes-based MR contrast agents, still there is a need for identifying biocompatible, tunable, functionalizable nanoparticles with high sensitivity and features like a switchable contrast known from CEST agents.

In this context, usage of nanoparticles as contrast agents for hp ^129^Xe MRI are emerging at preclinical stage in order to accomplish images of different cell types at relatively low labeling concentrations (∼nM–pM). Conversely, in clinics and clinical trials, hp ^129^Xe is often used to study declining lung parameters in humans during, e.g., cystic fibrosis, COPD, and asthma [180]. The translation of the potential of ^129^Xe HyperCEST MRI contrast agents from bench to bedside is in progress. The first *in vivo* application showed the feasibility based on the Xe host CB[6] [53] but clearly illustrated the need for better sensitivity since the injection concentration of the agent was in the millimolar range (see Section 5.3). Such translation is not straightforward and has to overcome certain experimental challenges, e.g., Xe hyperpolarization levels and accelerated MR encoding. The in situ formation of the host-guest complex is a great advantage of this technique. Unlike MRI with other hp tracers, ^129^Xe HyperCEST MRI can be performed in two steps: First, the administration of the Xe hosts into systemic circulation independent from Xe delivery leaves sufficient time for them to bind to molecular targets and for wash out of the unbound hosts. This is followed by a convenient flexibility in terms of hp ^129^Xe delivery, either via inhalation, or by *i.v.* administration of dissolved hp Xe [181]. The limitation of the CB[6]-based approach illustrates that simple hosts for individual Xe atoms like in the original biosensor approach will probably not be sufficient to cause a measurable HyperCEST effect within the limited hp lifetime at interesting concentrations (low *μ*M at maximum). Moreover, the chemical shift differences proposed in the original paper by Spence et al. [18] to distinguish between bound and unbound sensor will presumably remain unresolved under *in vivo* conditions (at least for most of the currently known sensors). This is not an issue as long as specific uptake through well-designed targeting units is ensured. A strong incorporation of nanoparticle design is needed to achieve scaffolds with many synthetic hosts like CryA or to advance the work with larger host structures for hundreds to thousands of Xe atoms like in nanodroplets or bacterial GVs.

Pursuing this road to *in vivo* studies, successful translation of this unprecedented potential may be challenged by toxicity issues emerging from the nanoparticles rather than Xe itself. The cytotoxicity effects of different Xe hosts (synthetic and biogenic ones) on various cell types *in vitro* and *in vivo* have been investigated to some extent but more studies are required. Conversely, higher concentrations of Xe (v/v ∼80%) are known to cause only an anesthetic effect, presumably by affecting the cell surface receptors and certain cellular signaling pathways [182]. To promote *in vivo* investigations, the hyperpolarization of ^129^Xe should be preserved as high as possible during the delivery step. To do so, hp ^129^Xe can be mixed with saline solution and was investigated after mixing with a human blood sample for studying the relaxation behavior, exchange, and binding of Xe to different components (e.g., proteins and paramagnetic moieties). A peak at ∼32 ppm downfield from dissolved Xe was observed for Xe bound to red blood cells (RBCs) due to the close proximity of the heme group [183]. The change in the relaxation behavior of hp Xe upon inhalation is also an important aspect because inhalation serves as another route for Xe administration. The relatively long *T*_1_ relaxation time of hp Xe is reduced upon admixing with O_2_ where the latter is required for ventilation purposes during inhalation-based Xe delivery. Therefore, the exposure time of Xe to paramagnetic O_2_ should be minimal. After inhalation, the O_2_ concentration in the lung and the blood will also contribute to the faster relaxation of Xe nuclei, thereby limiting the detectable Xe signal in different tissues [26].

In most studies, a lower Xe percentage (e.g., 2 or 5%) in the gas mixture is used for the SEOP process with polarization levels of 20–30%. Improvements can be achieved by enhancing the ^129^Xe polarization through optimizing the optical cell length, gas flow, or by using isotopically enriched Xe, respectively. Combination with currently already available lung imaging in humans [184] could make the application of, e.g., Xe-binding nanoemulsions a powerful tool for the detection of lung cancer. Generation of targeted nanoparticles for various pathologies might also rely on passive targeting facilitated by the EPR effect leading to its tumor accumulation via the leaky tumor vasculature. Interestingly, the capabilities of PFC nanoemulsions for labeling tumors have been reported to depend upon such nonspecific accumulation over time [185, 186]. To proceed with the *in vivo* testing of nanoemulsion-based HyperCEST agents, the following considerations should be dealt with:

- Accessibility of targeted Xe hosts to circulating and diffusing Xe
- Time scale and dynamics of host-Xe interactions in relation to *T*_1_ relaxation of the ^129^Xe bulk signal
- Size of targeted pathologies compared to achievable dissolved-phase Xe MR image resolution
- Changes in RF sensitivity due to usage of larger coil and sample displacements, etc.

It was reported that slowing down the Xe exchange might enhance the contrast generated from nanoemulsion hosts with the fast exchange behavior. Thus, an option is PFC nanoemulsion incorporation into different surfactants [110]. Considering the aforementioned ^129^Xe HyperCEST *in vivo* challenges and the potential solutions available to overcome different issues linked to Xe and its host, preclinical molecular imaging using hp ^129^Xe and its translation into clinics clearly seems feasible.

### 5.3. Future Directions

Regarding the (pre-)clinical translation of ^129^Xe HyperCEST, the overall approach should lead to a scenario where a high local concentration of hp ^129^Xe can be achieved *in vivo* such that molecular imaging of different biological targets will be performed at ease. In this regard, nanoparticles could also play an important role as carriers for Xe and for increasing its local concentration. However, such nanoparticles should address certain aspects in order to become a valuable ^129^Xe carrier: (1) the structural pore or cavity size of nanoparticles should be adoptive for somewhat selective uptake of the noble gas and at the same time it should avoid inclusion of other endogenous molecules and (2) the nanoparticles must remain as stable colloidal suspensions without any undesired agglomeration during the *in vivo* investigation [187]. To confer better pharmacokinetic profile and biodistribution to such nanoparticles, polyethylene glycol (PEG) polymers can be installed on the nanoparticle surface. Mostly, PEGylation is performed for metallic and Si nanoparticles as they are prone to aggregation without any protective coating. Introduction of the PEG polymers on nanoparticles is also known to evade protein adsorption and platelet adhesion onto its surface [188].

To this end, the preclinical translation of silicalite nanoparticles as targeted ^129^Xe contrast agent was attempted *in vitro* using hp ^129^Xe NMR and *in vivo* biodistribution using *γ* scintigraphy. These silicalite nanoparticles (MFI-Si_96_O_192_) are known to possess a pore size (0.5–0.6 nm) that is well suited for hosting Xe with a diameter of 0.43 nm. The ^129^Xe NMR of silicalite nanoparticles in D_2_O indicated peaks at 7 ppm (Xe@gas), 196 ppm (Xe@solution), and 120–140 ppm (Xe@pores), respectively. The Xe relaxation time *T*_1_ inside the silicalite nanoparticle pores was measured as 30 s. The nanoparticles were silanized, followed by introduction of semicarbazide linkers. Finally, they were tethered to PEG polymers that provided the anchors for DTPA bis-anhydride necessary for complexation of an ^111^In ion as *γ* emitter. Additionally, either a peptide for targeting blood clots or a control peptide was attached to semicarbazide linkers available on the silicalite nanoparticle surface. ^129^Xe NMR spectra of nonspecific nanoparticles (control peptide + PEGylation) in H_2_O : D_2_O (95 : 5 v/v) at varying xenon/particle ratio resulted in a broad peak for Xe@pores at 120–160 ppm, and the peak maximum shifted to 145 ppm upon increasing Xe/particle concentration. *T*_1_ of Xe encapsulated in micropores of such nonspecific nanoparticles was even longer (41 s) compared to naked silicalite nanoparticles. Although silicalite nanoparticles targeting blood clots were synthesized, only the nonspecific counterpart was utilized to study its biodistribution. The *γ* scintigraphy results indicated that ^111^In complexed in silicalite nanoparticles was taken up by the liver and elimination appeared to be slow [187]. This study shows the feasibility of such ^129^Xe delivery for *in vivo* translation. However, it currently lacks important details such as pharmacokinetics, complete biodistribution, immune response, *in vivo* toxicity, and demonstration of a targeted molecular imaging approach with these silicalite nanoparticles.

Recently, a macrocyclic-based nanosystem was evaluated as a potential contrast agent for *in vivo*^129^Xe HyperCEST NMR and MRI. A preliminary investigation of Sprague-Dawley (SD) rats was reported after intravenously injecting them with unfunctionalized CB[6] at millimolar concentrations [53]. The SD rats were ventilated using a 80% Xe/20% O_2_ gas mixture, followed by 30 min biodistribution of CB[6] prior to ^129^Xe-based MRS and HyperCEST image acquisition. The MR spectrum of the rat abdomen indicated three peaks at 184 ppm (fat), 192.5 ppm (lung parenchyma), and 207 ppm (blood) in addition to the Xe@gas peak referenced at 0 ppm. A ^129^Xe HyperCEST response was observed for saturating CB[6] at 123.4 ppm which led to a 22% reduction in signal intensity compared to an off-resonance control spectrum. Contrarily, a HyperCEST spectrum from brain tissue indicated 55% reduction in signal intensity compared to its reference spectrum. The spectrum from the rat head indicated four peaks at 185 ppm (muscle), 191 ppm (white matter), 193 ppm (grey matter), and 205 ppm (red blood cells), respectively [189, 190]. The Xe carrier was localized by overlaying the *in vivo*^129^Xe HyperCEST map on the anatomical ^1^H MR images (see Figure 4(d)). Interestingly, off-resonance MR images indicated Xe distribution at highly perfused areas, including the brain, aorta, liver, kidney, lungs, and heart. The nonspecific localization of CB[6] in the brain, heart, lungs, liver, aorta, and kidneys was supported by comparing the on-resonant and off-resonant ^129^Xe HyperCEST images and by a control experiment in which no noticeable ^129^Xe HyperCEST signal was observed in the brain after PBS injection. Although this study appears promising, still there is a need for further improvements (e.g., Xe carrier functionalization and MRI sequence optimization) such that high sensitivity of Xe biosensors can be translated for different ^129^Xe-based molecular imaging applications [53] that are not feasible with existing contrast agents.

Although aforementioned *in vivo*^129^Xe HyperCEST attempts are prompting towards the direction of (pre-)clinical translation, still there are additional experimental challenges that remain unaddressed. This currently limits the depth of this otherwise expanding field. While many sensor types have been demonstrated *in vitro*, their application in live animals requires the combination of recent progress from the areas of production of hp Xe, the smart nanoparticle design, and the very latest MRI acquisition protocols for hp nuclei. Regarding the production of hp Xe, another factor of (only) 3–5 can be gained in sensitivity since many setups achieve already 20–30% polarization without too much effort. Regarding promising nanoparticle approaches, further characterization studies are needed: Firstly, the toxicity studies of the synthetic nanocarriers are incomplete—both in cells and in living organisms. Secondly, the colloidal stability of nanoparticles *in vivo* and their potential to selectively avoid the nonspecific inclusion complexation of endogenous molecules are also a concern. The plasma protein interaction and corona formation should be minimized such that the nanoparticles are not taken up quickly by the resident and circulating macrophages leading to a passive accumulation in the reticuloendothelial systems (e.g., liver and spleen). Extended circulation time of nanoparticles without agglomeration and complete clearance from the body are also a prerequisite for having a potential ^129^Xe HyperCEST biosensor. Such requirements might be fulfilled by using biogenic nanoparticles as platforms for *in vivo*^129^Xe HyperCEST MRI. In this regard, gas vesicles (GVs) appear as promising candidates for preclinical studies since genetically encoded hosts can process a higher Xe payload compared to any other synthetic carrier, e.g., PFOB nanodroplets. Recently, the potential of GVs was demonstrated by utilizing them as acoustic pressure sensors [191] that enabled acoustic contrast manipulation in cells. There is also experience with their application as *in vivo* contrast agents for optical coherence tomography (OCT) in detecting the mouse retina *in vivo* [192], as micron-scale bubbles for targeting the cell surface receptors (*in vitro*, *in cellulo*, and *in vivo*) [193], and as acoustic reporter genes for monitoring different mammalian cells *in vivo* [151], respectively. Typically, the nanosize of GVs (e.g., *Mega* GVs) might foster their application as contrast agents for targeting different targets outside the bloodstream, e.g., tumors [194]. Similar to other imaging approaches, PEGylation of GVs surface might be necessary to achieve an increased circulation time for reducing clearance by organs and for decreasing the immunogenic response [195]. The *in vivo* safety of GVs in potential preclinical applications requiring a repeated administration must be addressed as well.

The *in vivo* translation conditions for ^129^Xe HyperCEST MRI has been predicted using a pharmacokinetic modeling which indicated that a repeated Xe inhalation and low flip-angle excitations are necessary for continuous imaging and on-resonance saturation [54]. A signal reduction of 73% was predicted in GV-containing regions *in vivo*. Further, Xe concentrations known from different experiments shall be sufficient to detect 400 pM GVs using ^129^Xe HyperCEST schemes adopted to *in vivo* requirements. The modeling also supports the feasibility of HyperCEST imaging of GV-based reporters localized in the brain and different vascularized organs. However, a mechanistic understanding of the GV assembly process is important since heterologous expression in target cell types might be critical for utilizing GVs as *in vivo* imaging agents [196]. The phenotypic diversity of GVs could be exploited to yield various GVs with material properties optimized for different imaging modalities. Taken together, the perspectives for combining the HyperCEST method with nanotechnology approaches are promising for implementing Xe biosensors. Their high sensitivity can address previously inaccessible molecular targets, and this could eventually significantly expand the role of MRI in precision diagnostics.
